# Immunophenotypic Features of T-Lymphocyte Distribution Across Histological and Molecular Subtypes of Serous Ovarian Neoplasms: A Retrospective Immunohistochemical Study

**DOI:** 10.3390/biomedicines14102275

**Published:** 2026-10-08

**Authors:** Grigory Demyashkin, Sofia Dorfman, Aleksandra Asaturova, Aleksandra Rogozhina, Alina Badlaeva, Vladislav Holdeenko, Vladimir Shchekin, Nina Kulchenko, Tamara Kononenko, Matvey Vadyukhin, Nazim Amirov, Tatyana Borovaya, Petr Shegai, Andrey Kaprin

**Affiliations:** 1 Department of Digital Oncomorphology, National Medical Research Centre of Radiology, 2nd Botkinsky Pass., 3, 125284 Moscow, Russia; dorfmansofia@yahoo.com (S.D.); dr.shchekin@mail.ru (V.S.); lokoport@mail.ru (T.K.); vma20@mail.ru (M.V.); amirovnazim@gmail.com (N.A.); tbor27@yandex.ru (T.B.); dr.shegai@mail.ru (P.S.); kaprin@mail.ru (A.K.); 2 Laboratory of Histology and Immunohistochemistry, Institute of Translational Medicine and Biotechnology, I.M. Sechenov First Moscow State Medical University ( Sechenov University), Trubetskaya St., 8/2, 119048 Moscow, Russia; 3 National Medical Research Center for Obstetrics, Gynecology and Perinatology Named After Academician V.I. Kulakov, Oparina St., 4, 117513 Moscow, Russia; a.asaturova@gmail.com (A.A.); doc.rogozhina@gmail.com (A.R.); alinamagnaeva03@gmail.com (A.B.); 4 Research and Educational Resource Center for Immunophenotyping, Digital Spatial Profiling and Ultrastructural Analysis Innovative Technologies, Peoples’ Friendship University of Russia (RUDN University), Miklukho-Maklaya St., 6, 117198 Moscow, Russia; vlad.xoldeenko@mail.ru (V.H.); kle-kni@mail.ru (N.K.); 5 Department of Urology and Operative Nephrology, Peoples’ Friendship University of Russia (RUDN University), Miklukho-Maklaya Str., 6, 117198 Moscow, Russia

**Keywords:** ovarian cancer, serous ovarian tumors, CD3, CD4, CD8, FOXP3, immunohistochemistry, T lymphocytes

## Abstract

**Background/Objectives**: Serous ovarian neoplasms comprise biologically distinct entities with substantial differences in clinical behavior and molecular characteristics. Comparative data on the quantitative and spatial distribution of T-cell populations across the spectrum of serous ovarian neoplasms, particularly borderline ovarian tumors (BOT) and serous cystadenomas (CA), remain limited. The aim of this study was to compare the quantitative and spatial distribution of CD3+, CD4+, CD8+, and FOXP3+ cells across serous ovarian neoplasms and to assess their associations with selected molecular characteristics. **Methods**: This retrospective, single-center observational study included 227 patients with high-grade serous carcinoma (HGSC; *n* = 108), low-grade serous carcinoma (LGSC; *n* = 49), BOT (*n* = 16), and CA (*n* = 54). The primary endpoint was the density of CD3+, CD4+, CD8+ and FOXP3+ immunopositive cells per mm^2^, evaluated by immunohistochemistry and digital morphometry. Spatial distribution, differences across early pT substages, the CD8+/FOXP3+ ratio, and associations with selected molecular characteristics were additionally assessed. Age-adjusted generalized linear models were used to evaluate whether differences among histological groups were independent of patient age. **Results**: Compared with CA, HGSC showed an 18.58-fold higher CD3+ cell density and a 9.07-fold higher FOXP3+ cell density (both *p* < 0.001). Histological type remained significantly associated with CD3+, CD4+, CD8+, and FOXP3+ cell densities after adjustment for patient age (all *p* < 0.001), whereas age itself was not independently associated with any of the four markers (all *p* > 0.05). CD3+ cells were predominantly distributed in peritumoral areas, whereas HGSC also demonstrated diffuse intratumoral and stromal infiltration. Comparatively high T-cell densities were observed in BRCA1/2-mutated HGSC and HRD-positive subgroups. No statistically significant differences in immunopositive cell densities were detected across the analyzed pT1 substages. HGSC showed the lowest CD8+/FOXP3+ ratio (Me 1.18 [1.06–1.34]). **Conclusions**: Serous ovarian neoplasms demonstrate distinct histology-associated patterns of T-cell infiltration and spatial distribution, with additional heterogeneity across selected molecular subgroups. These differences remained evident after adjustment for patient age. Their functional and prognostic significance requires further validation using expanded immunophenotypic panels, multiplex approaches, and longitudinal clinical outcomes.

## 1. Introduction

Ovarian cancer (OC) is one of the most aggressive malignancies of the female reproductive system and is characterized by high mortality. According to the World Health Organization (WHO), approximately 325,000 new cases of OC and 205,000 deaths from the disease are reported worldwide annually [1]. These high rates are largely attributable to delayed diagnosis due to the asymptomatic and nonspecific course of the disease at its early stages [2]. The overall 5-year survival rate of patients with OC is 52–54% [3]. The main risk factors for OC include menopausal age, a family history of this type of cancer, and late childbirth (after the age of 35 years) [1].

Depending on the degree of differentiation, two histological subtypes of serous ovarian carcinoma are distinguished: high-grade serous carcinoma (HGSC) and low-grade serous carcinoma (LGSC). HGSC predominates, accounting for approximately 70% of cases, whereas LGSC accounts for only about 5%. These tumors differ in their morphological and molecular characteristics, biological behavior, and clinical course [4,5].

Serous borderline ovarian tumors (borderline ovarian tumor, BOT) are of particular scientific interest because this type of serous neoplasm fits most closely within the concept of stepwise carcinogenesis.

Another benign serous epithelial neoplasm of the ovary is cystadenoma (CA), which accounts for approximately 40% of benign tumors of the female reproductive system. KRAS and BRAF alterations are not considered characteristic molecular features of CA [6].

Although a pathway of LGSC development from BOT has been described, these types of neoplasms do not always represent sequential stages of a single malignant transformation process. In contrast, HGSC develops through an independent molecular pathway of carcinogenesis [4,5,6].

The nonspecific nature of the clinical manifestations of serous ovarian neoplasms, including benign lesions, complicates their timely verification and diagnosis [1]. A comprehensive assessment of these histological types within a single comparative study may allow changes in the local immune microenvironment to be traced across different phenotypes of serous ovarian neoplasms.

The tumor microenvironment (TME) plays one of the key roles in tumor progression across cancers of various anatomical sites. In addition, the microenvironment is an important parameter for assessing tumor malignancy and may serve as a marker of sensitivity to antitumor therapy [7,8]. The TME consists of immune cells, stromal and vascular components, and extracellular components surrounding the tumor. The principal cellular constituents of the tumor immune microenvironment (TIME) include various T-lymphocyte subpopulations, NK cells, macrophages, mast cells, dendritic cells, and others [9]. The stromal component is represented by cells of the fibroblastic lineage, whereas the vascular component is represented by somatic capillaries.

Recent studies have demonstrated pronounced immunosuppressive features of the immune microenvironment in ovarian cancer. At the same time, some authors have reported associations between characteristics of the TIME and the efficacy of antitumor immunotherapy [10,11].

Tumor-infiltrating lymphocytes (TILs) play a fundamental role in cell-mediated immune surveillance. This heterogeneous cell population arises as a result of lymphocyte extravasation from the microcirculatory bed and their directed migration into the tumor. Lymphocytic infiltration involves both stromal and directly intraepithelial areas of ovarian cancer. Specific TIL subpopulations are identified by assessing the expression of their respective clusters of differentiation.

The pan-T-cell marker CD3 is widely used for the overall assessment of the T-cell component of TILs. Its detection enables quantitative assessment of the total T-cell population but does not allow determination of their activation status or cytotoxic potential. CD4 is expressed by monocytes, macrophages, and regulatory T lymphocytes; however, it is primarily regarded as a marker of T-helper cells [12]. Previous studies by other authors have demonstrated heterogeneity of this subpopulation in patients with HGSC [13]. CD8 is routinely used as a marker for the identification of cytotoxic T lymphocytes. Other authors have reported associations between CD8+ cell density and clinical outcomes in patients with HGSC [10,14].

In the context of ovarian cancer, a comprehensive assessment of the TIME also requires evaluation of an additional lymphocyte population—FOXP3+ cells. Regulatory T cells expressing this marker are associated with the formation of an immunosuppressive microenvironment through the secretion of TGF-β, IL-10, and IL-35, thereby promoting effective immune evasion by OC. However, FOXP3 expression alone does not directly establish the suppressive function of individual cells and may also be detected in transiently activated conventional T lymphocytes [14,15].

Quantitative analysis of CD3+, CD4+, CD8+, and FOXP3+ cells, followed by comparison of the obtained results across histological types of serous ovarian neoplasms, is of particular interest for identifying characteristic features of the TIME associated with different serous tumor phenotypes.

Nevertheless, the role of TILs in OC remains controversial in the literature. Furthermore, comparative data on the distribution of T-cell populations in other serous ovarian neoplasms, particularly BOT and CA, remain limited. Such data, however, may be highly relevant for understanding the mechanisms of carcinogenesis and tumor progression, as well as for the development of novel immunotherapeutic approaches. Of additional interest is the investigation of associations between cell density and available molecular characteristics, including BRCA1/2 status, homologous recombination deficiency (HRD), alterations in TP53, KRAS, and BRAF, and the Ki-67 index, given their relevance to the corresponding phenotypes.

Systematization of data on lymphocyte marker profiles and localization across different phenotypes of serous ovarian neoplasms in the context of the hypothesis of tumor transformation and progression may contribute to a deeper understanding of the molecular and biological mechanisms of ovarian carcinogenesis and provide a fundamental basis for their further investigation and the subsequent identification of potential prognostic markers, as well as novel targets for personalized therapeutic approaches, including antigen-specific antitumor therapy.

Therefore, the aim of the present study was to determine and subsequently perform a comparative analysis of both the quantitative and spatial characteristics of CD3+, CD4+, CD8+, and FOXP3+ cell distribution across serous ovarian neoplasms, as well as to investigate their associations with selected molecular characteristics.

## 2. Materials and Methods

### 2.1. Study Design and Setting

This retrospective, singlecenter, observational, cohort study was conducted from 2020 to 2025 at the Department of Oncogynecology and Department of Digital oncomorphology of the National Medical Research Radiological Center of the Ministry of Health of the Russian Federation. Potential patient cases were initially identified through a search of the electronic medical record system using International Classification of Diseases, 10th Revision (ICD-10) codes corresponding to the primary diagnosis C56—Malignant neoplasm of ovary, D39.1—Neoplasm of uncertain or unknown behavior of ovary, and D27—Benign neoplasm of ovary.

The study was approved by the Local Ethics Committee of the National Medical Research Radiological Center (protocol No. 19e, dated 2 March 2020). Owing to the retrospective and non-interventional nature of the study, the requirement for written informed consent was waived by the ethics committee. All biological specimens and patient-related data were handled in accordance with the applicable guidelines from human biobanks and genetic research databases, with strict measures implemented to ensure anonymization, confidentiality and secure data storage. The study was conducted in accordance with the principles of the Declaration of Helsinki, and the reporting of the study followed the STROBE (Strengthening the Reporting of Observational Studies in Epidemiology) guidelines.

### 2.2. Patient Selection

**Figure 1:**
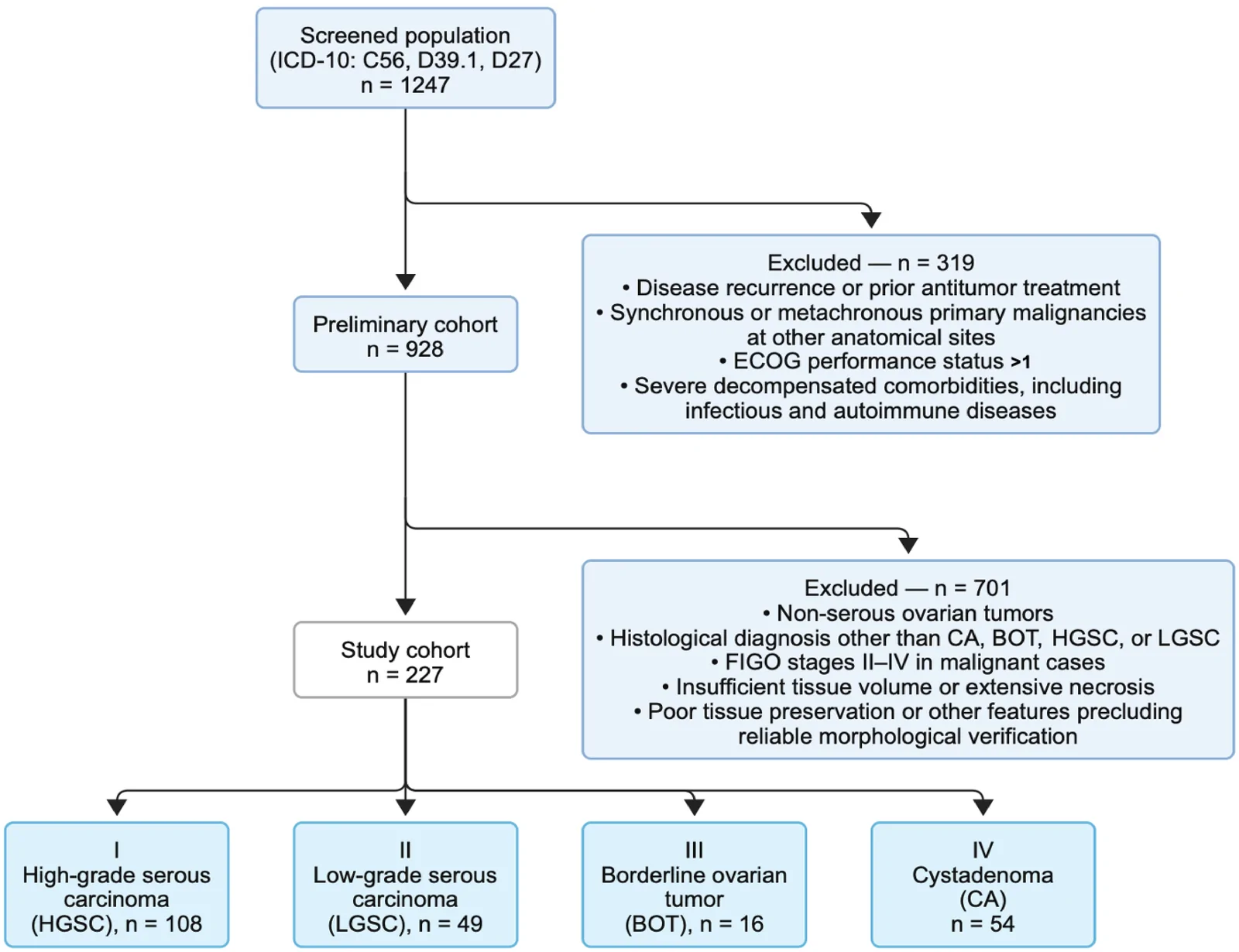
Groups of patients.

When analyzing the clinical data, the following parameters were taken into account: age, histological type, and mutational status of the subjects (Figure 1).

*Inclusion Criteria.* Primary histologically confirmed serous ovarian neoplasms, namely serous cystadenoma (CA), borderline ovarian tumor (BOT), high-grade serous carcinoma (HGSC), and low-grade serous carcinoma (LGSC); an Eastern Cooperative Oncology Group (ECOG) performance status of 0–1; and the availability of representative archival tissue fixed in neutral-buffered formalin and embedded in paraffin (FFPE).

The histological type of the tumors was determined according to the criteria of the 5th edition of the World Health Organization classification (WHO Classification of Tumours: Female Genital Tumours, 2020). Staging was performed according to the 2014 classification of the International Federation of Gynecology and Obstetrics (FIGO), which is also integrated into the 8th edition of the AJCC classification. For a more detailed analysis of local tumor progression at the early stages of carcinogenesis, HGSC and LGSC cases were additionally stratified according to pT substages. This approach enabled the assessment of immunological changes in the context of the depth of invasion and spatial distribution. pT staging was not applied to CA and BOT because of their lack of invasive potential.

*Exclusion Criteria.* The presence of other disease stages (II, III, or IV) at the time of initial diagnosis in patients with malignant neoplasms; previously diagnosed disease recurrence or any prior antitumor treatment (surgical, pharmacological, or radiation therapy) for the respective disease before inclusion in the study; synchronous or metachronous multiple primary malignant neoplasms at other anatomical sites; non-serous ovarian tumors (germ cell, stromal, mucinous, and other tumors); insufficient tissue volume, extensive necrosis, poor tissue preservation, or other features precluding reliable morphological verification; and the presence of severe decompensated comorbidities, including infectious and autoimmune diseases, that could substantially affect overall survival and the feasibility of specific antitumor treatment.

Because this was a retrospective study based on archival material, no a priori sample-size calculation was performed. The final cohort was determined by the availability of eligible archival cases meeting the predefined inclusion and exclusion criteria during the study period. All eligible cases were included in the analysis. Analyses involving small molecular subgroups were considered exploratory.

### 2.3. Outcome Measures

The primary endpoint was the density of CD3+, CD4+, CD8+ and FOXP3+ TILs per mm^2^ in serous ovarian neoplasms, evaluated by immunohistochemistry and digital morphometry. Secondary endpoints included the spatial distribution of these cells (peritumoral versus diffuse) and differences in TIL density across early pathological stages (pT1a-pT1c3). Additionally, we assessed the associations between the immune microenvironment and key molecular profiles (BRCA 1/2, TP53, KRAS/BRAF mutations and HRD status). Finally, the CD8+/FOXP3+ ratio was calculated individually for each patient to evaluate the balance of the local immune response.

### 2.4. Molecular Genetic and Morphological Block

#### 2.4.1. Targeted Molecular Analysis and Homologous Recombination Deficiency (HRD) Profiling

Venous blood samples were obtained from the patients and subsequently analyzed for germline *BRCA1/2* gene mutations using the real-time polymerase chain reaction (real-time PCR) method (DTPrime4; DNA-Technology, Moscow, Russia). The specific mutations investigated in the *BRCA1* gene included 5382insC (insertion of C at position 5382); 4153delA (deletion of A at position 4153); 300T>G (substitution of T to Gat position 300); 3819deIGTAAA (deletion of GTAAA at position 3819); 2080delA (deletion of A at position 2080); 185delAG (deletion of AG at position 185); and 3875delGTCT (deletion of GTCT at position 3875). For the *BRCA2* gene, the 6174 delT mutation (deletion of T at position 6174) was evaluated.

Furthermore, somatic mutation analysis of the *TP53*, *KRAS*, and *BRAF* genes was performed using genomic DNA extracted from archival formalin-fixed paraffin-embedded (FFPE) tumor tissues. The analysis targeted highly recurrent mutational hotspots, including missense mutations within exons 5–8 of the *TP53* gene (the DNA-binding domain), *KRAS* variants at codons 12, 13, and 61, and the *BRAF* V600E (c.1799T>A) mutation.

Finally, the HRD status was assessed to identify HRD-positive (HRD+) cases. The HRD evaluation was carried out using a targeted next-generation sequencing (NGS) panel to determine the genomic instability score based on loss of heterozygosity (LOH), telomeric allelic imbalance (TAI), and large-scale state transitions (LST).

#### 2.4.2. Histologic Research

Fragments of serous ovarian neoplasms were fixed in 10% neutral-buffered formalin and embedded in paraffin. Serial 2 μm thick sections were obtained from the resulting paraffin blocks and stained with hematoxylin and eosin (H&E). Histological analysis was performed using a Leica DM2000 light microscope with digital microphotography capabilities (Leica Microsystems, Wetzlar, Germany) in accordance with standard histopathological criteria. For this retrospective study, all stained sections were independently re-evaluated by two experienced pathologists who were blinded to the original pathology reports and clinical data. Any discrepancies in interpretation were resolved through joint discussion to reach a consensus.

#### 2.4.3. Immunohistochemical (IHC) Assay

For the immunohistochemical study, the sections were deparaffinized and rehydrated. Heat-induced epitope retrieval was then performed using Universal HIER antigen retrieval reagent (ab208572, Abcam; Cambridge, UK) for 20 min. Endogenous peroxidase activity was blocked with 3% hydrogen peroxide. Sections were then incubated overnight at 4 °C with the following primary rabbit monoclonal antibodies: anti-CD3 (Abcam; clone SP162, ab135372, dilution 1:150, Cambridge, UK), anti-CD4 (Abcam; clone CAL4, ab237722, dilution 1:4000, Cambridge, UK), anti-CD8 (Abcam; clone EPR21769, ab217344, dilution 1:2000, Cambridge, UK), anti-FOXP3 (Abcam; clone EPR22102-37, ab215206, dilution 1:500, Cambridge, UK), and anti-Ki-67 (Abcam; clone SP6, ab16667, dilution 1:200, Cambridge, UK). For signal detection, the HiDef Detection™ HRP Polymer System (Cell Marque, Rocklin, CA, USA) was applied according to the manufacturer’s instructions. The reaction was visualized using 3,3′-diaminobenzidine (DAB) chromogen, followed by counterstaining with Mayer’s hematoxylin and mounting.

Positive controls: Human tonsil tissue—rich in diverse and topographically distinct T-lymphocyte populations and highly proliferative germinal centers—served as the positive control for CD3, CD4, CD8, FOXP3, and Ki-67. Negative controls: Adjacent sections were processed by omitting the primary antibodies and using isotype-matched rabbit IgG instead, to evaluate nonspecific background staining and secondary system cross-reactivity. The staining patterns in the control tissues aligned with the expected cellular localizations, thereby validating the specificity and reliability of the assay for evaluating the experimental serous ovarian neoplasm samples.

### 2.5. Morphometric Study

Immunostained slides were digitized using a Leica Aperio AT2 scanner. To quantify CD3+, CD4+, CD8+, and FOXP3+ TILs, 3–5 representative hotspots in both intratumoral and peritumoral regions were selected through low-magnification screening. Regions of interest (ROIs) strictly excluded necrosis, hemorrhage, and structural artifacts, including tissue folds, cautery, and non-representative edges. Regions exhibiting stromal remodeling, such as hyalinosis or fibrosis, were analyzed as distinct morphological variables. Image analysis was performed at 200× magnification baseline utilizing QuPath v0.7.0 [16]. The software’s optical density-based algorithms detected absolute immunopositive cell counts, standardizing them to cells/mm^2^. Concurrently, the Ki-67 labeling index was assessed using a 30% cut-off to classify the cohort into “Low” and “High” proliferation groups, based on previously published studies in epithelial ovarian neoplasms using this threshold to distinguish lower from higher proliferative activity [17,18]. For each case, the data obtained from the selected hotspots were averaged to generate a single aggregate value. Consequently, the patient rather than individual microscopic fields served as the independent unit for all statistical analyses.

### 2.6. Statistical Analysis

All statistical computations were carried out utilizing IBM SPSS Statistics version 29.0 (IBM Corp., Armonk, NY, USA). The Shapiro–Wilk test was applied to assess the normality of continuous variables, which are expressed as medians with interquartile ranges throughout the manuscript as Me [Q1–Q3] due to non-normal distribution. Differences across three or more independent groups were analyzed using the non-parametric Kruskal–Wallis test, followed by Dunn’s pairwise comparisons with Holm correction to control for the family-wise error rate. The Mann–Whitney U test was utilized strictly for comparisons between two independent groups. To assess whether differences in immunopositive cell densities among histological groups were independent of patient age, separate generalized linear models were fitted for CD3+, CD4+, CD8+, and FOXP3+ cell densities using a gamma distribution with a log link. Histological group was included as a categorical predictor, with CA as the reference category, and age as a continuous covariate. Adjusted density ratios are reported with 95% confidence intervals (CI). A two-sided *p*-value of <0.05 was considered statistically significant; *p*-values from Dunn’s pairwise comparisons were adjusted using the Holm method. Violin plots were generated using GraphPad version 11.0.2 (GraphPad Software, Boston, MA, USA), and forest plots of age-adjusted estimates were generated in RStudio version 2026.09.0 (Posit Software, PBC, Boston, MA, USA). The conceptual graphical abstract was created with BioRender.com.

## 3. Results

Patients with malignant HGSC dominated among the subjects (*n* = 108; 47.6%). Among benign neoplasms, CA was the most common (*n* = 54; 23.8%). BOT was the least represented group (*n* = 16; 7.0%). In the age structure, the youngest patients were those with CA, whose age was 43.6 ± 3.9 years. In contrast, patients with HGSC were represented by an older age group, where the average age was 59.7 ± 3.7 years. The average age of patients with borderline tumors was 45.3 ± 2.6 years. The HGSC group showed a distinct molecular profile characterized by frequent TP53 and BRCA1/2 alterations and predominantly high Ki-67 proliferative activity. In contrast, KRAS/BRAF alterations were mainly observed in LGSC and BOT and were rare in CA. HRD-positive status was identified across HGSC, LGSC, and BOT, whereas low Ki-67 proliferative activity predominated in LGSC and BOT (Table 1).

### 3.1. Histological Patterns

**Figure 2:**
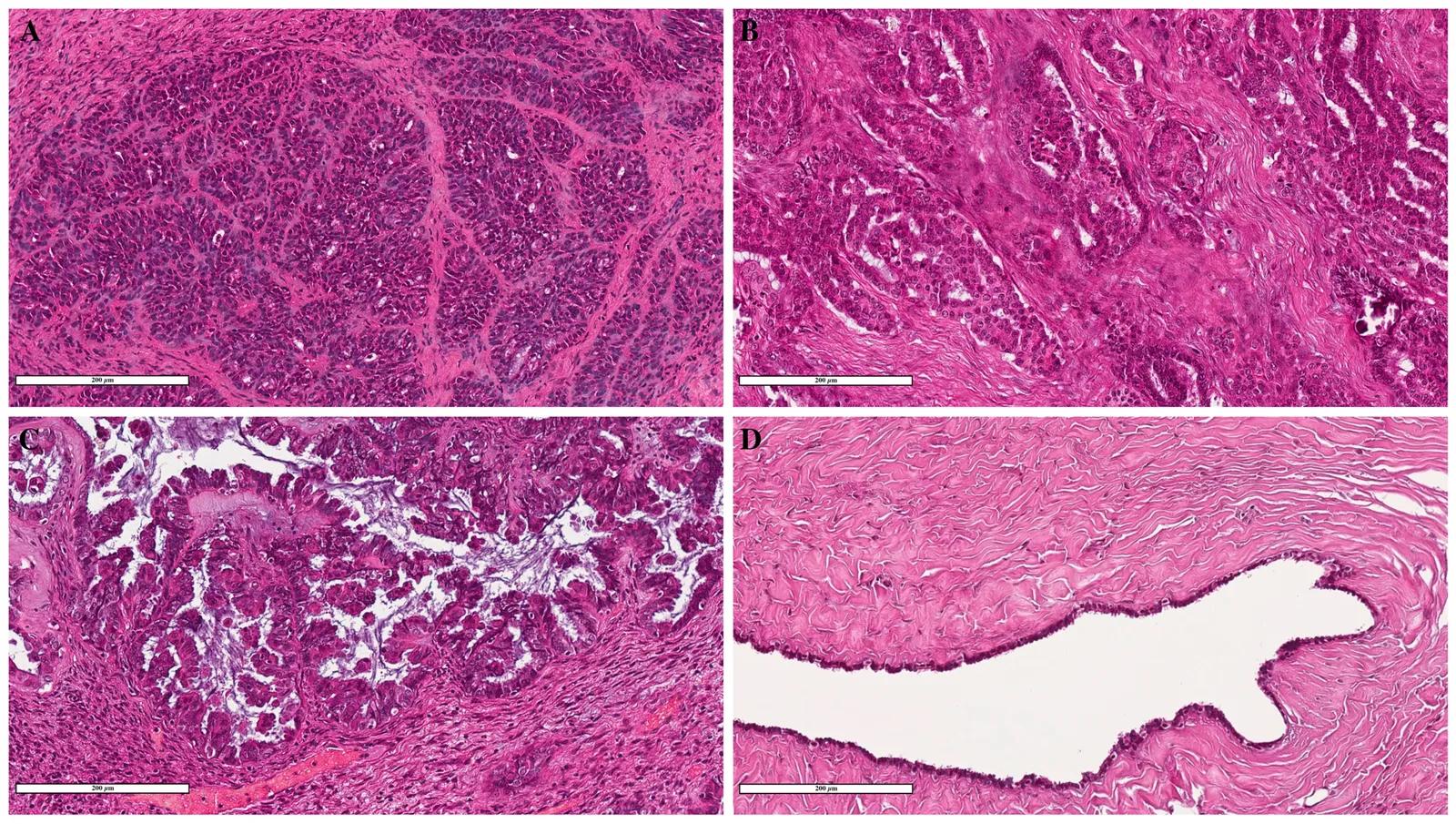
Histological (H&E) features in serous ovarian neoplasms: (**A**) HGSC, (**B**) LGSC, (**C**) BOT, (**D**) CA. Magnification ×200; scale bars = 200 µm.

Microscopically, the morphological picture of high-grade ovarian serous carcinoma were observed in patients of group I (*n* = 108). The tumor is characterized by pronounced nuclear polymorphism, high mitotic activity (including pathological mitoses), and a variety of patterns: solid fields, pseudocellular and cribriform structures, papillary formations, and areas of necrosis (Figure 2A). Histological examination of the preparations of patients from group II (*n* = 49) revealed the morphological features of low-grade serous ovarian carcinoma with stromal invasion. Most cancer cells had monomorphic nuclei and moderate atypia. Histoarchitecture was represented by small nests, papillae, or glandular structures; pathological mitotic figures were rare (Figure 2B).

In patients from group III (*n* = 16), histological examination revealed the morphological features of a serous borderline tumor, characterized by pronounced epithelial proliferation with the formation of papillary structures. Mitotic activity was low (Figure 2C).

Histological examination of specimens from patients in group IV (*n* = 54) revealed the morphological features of serous cystadenoma, represented by a cystic neoplasm lined with a single layer of cuboidal or columnar epithelium without papillary growths. Epithelial cells were small to medium-sized and had rounded nuclei with smooth contours and evenly distributed chromatin; nucleoli were rare and small. Psammoma bodies were observed in variable numbers in specimens from all groups (Figure 2D).

### 3.2. Immunohistochemical Assay

Serous ovarian neoplasms demonstrated a local inflammatory response of varying intensity, with differences in the extent of stromal and parenchymal involvement across histological groups.

Anti-CD3 antibodies were used to assess the level of T-lymphocyte infiltration in the peritumoral zone of ovarian serous neoplasms.

**Figure 3:**
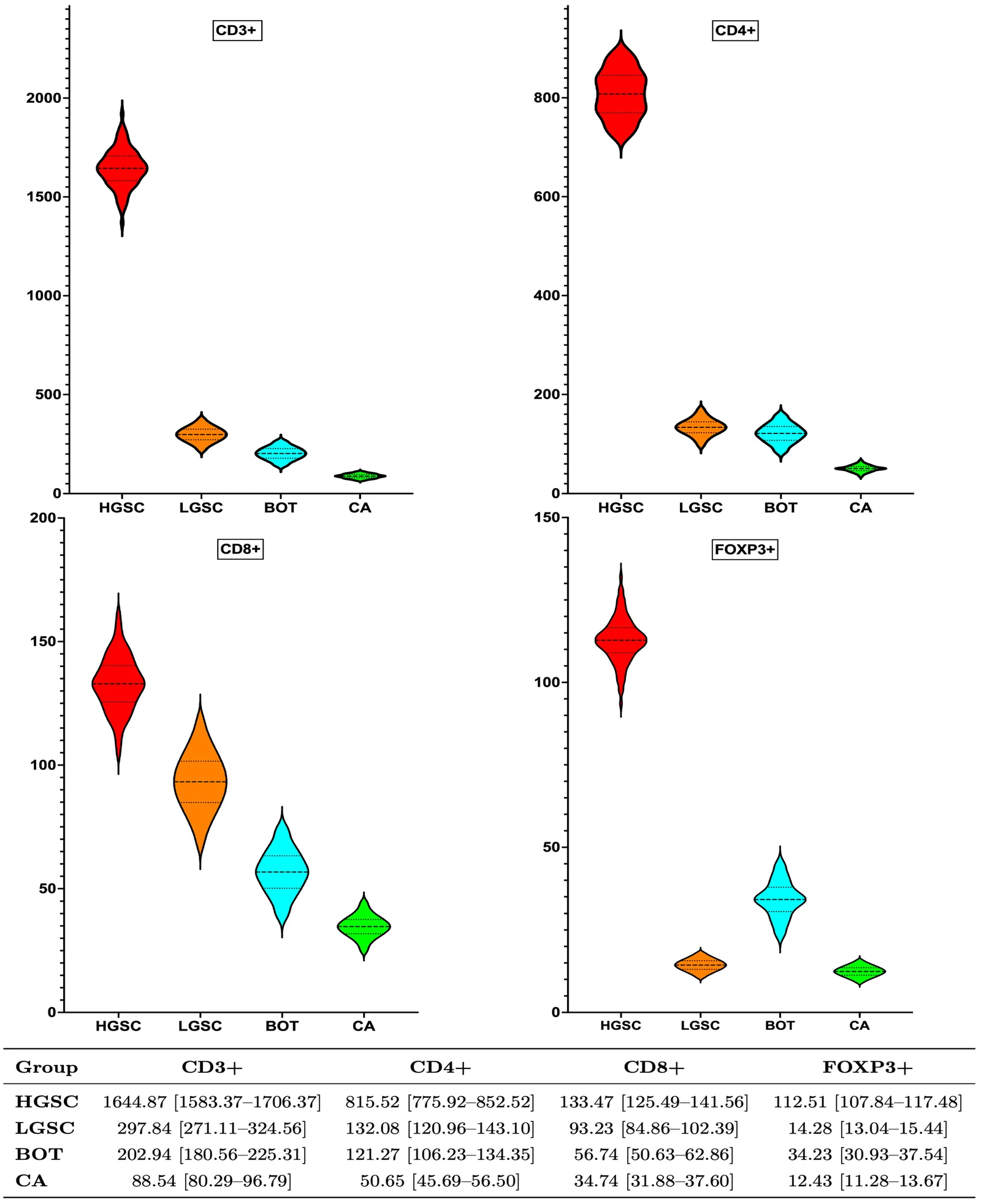
Density of CD3+, CD4+, CD8+, and FOXP3+ lymphocytes in the peritumoral zone according morphological type ovarian neoplasm. The x-axis represents study groups; the y-axis represents numerical cell density (number of immunopositive lymphocyte per 1 mm^2^). Within the violin plots, the central dashed lines represent the median, and the upper and lower dotted lines represent Q1 and Q3.

**Figure 4:**
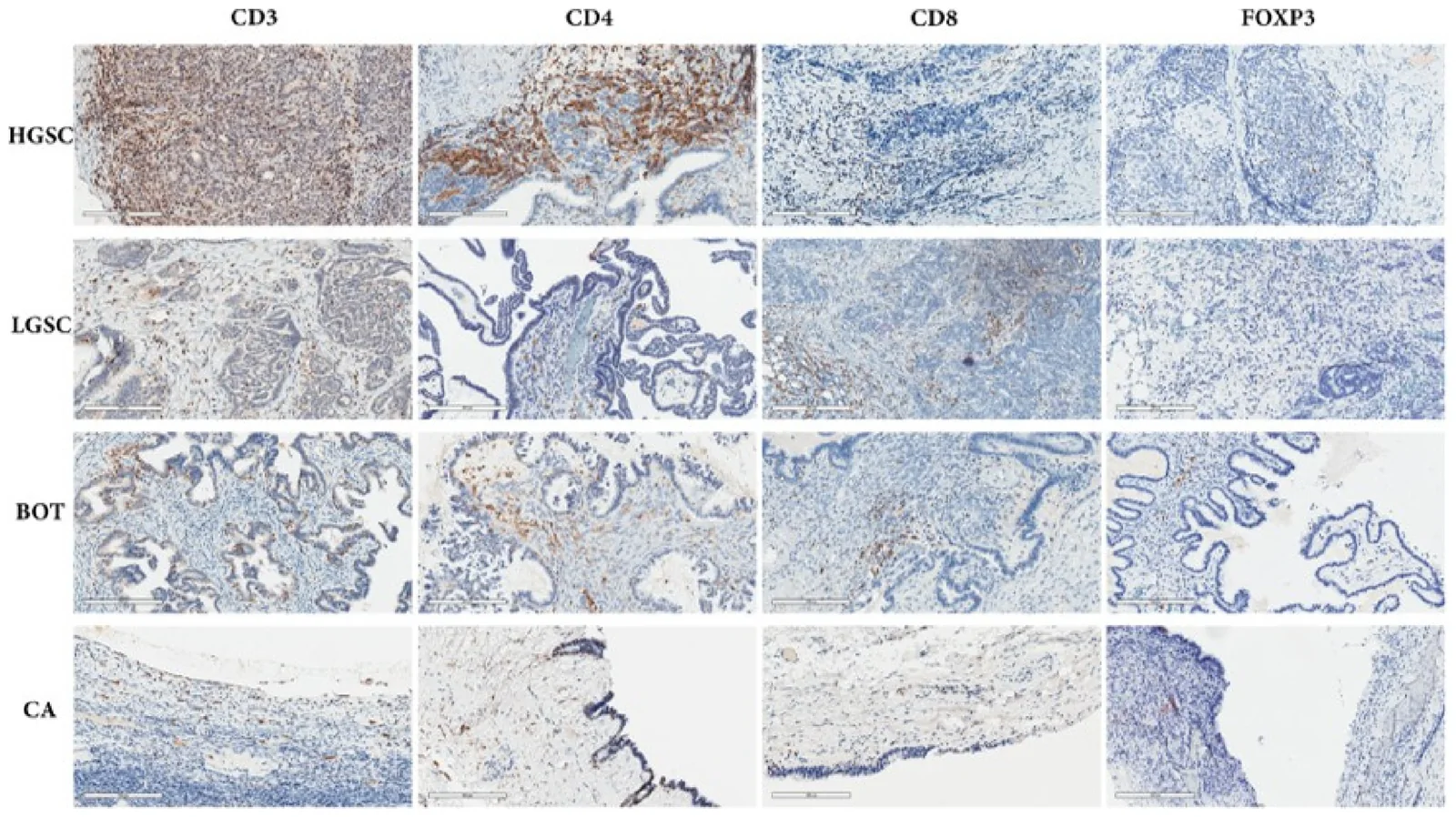
Immunohistochemical staining for CD3, CD4, CD8 and FOXP3 of serous ovarian neoplasms, magnification ×200; scale bars = 200 µm.

Immunohistochemical analysis revealed marked differences in CD3+ T-lymphocyte density among the histological groups, with the highest values observed in HGSC and the lowest in CA. Thus, in comparison with CA, CD3+ cell density was 2.29-fold higher in patients with BOT (*p* = 0.107), 3.36-fold higher in patients with LGSC (*p* < 0.001), and 18.58-fold higher in the cohort of patients with HGSC (*p* < 0.001). Qualitative assessment of CD3+ cell distribution showed predominant accumulation in the peritumoral regions, whereas HGSC additionally demonstrated diffuse infiltration involving both the stromal and intratumoral compartments. However, intergroup differences in the spatial distribution of CD3+ cells did not reach statistical significance (Figure 3 and Figure 4).

CD4 is a transmembrane glycoprotein expressed predominantly by T-helper lymphocytes but also by regulatory T cells and several other immune cell populations, including monocytes, macrophages, and dendritic cells. Quantitative analysis of CD4+ cell density demonstrated a 2.39-fold higher in BOT (*p* = 0.014), a 2.64-fold higher in LGSC (*p* < 0.001), and a 15.96-fold higher in HGSC compared with CA (*p* < 0.001) (Figure 3 and Figure 4).

CD8 is a transmembrane glycoprotein involved in the recognition of MHC class I-associated antigens together with the T-cell receptor and is commonly used as a marker of cytotoxic T lymphocytes, while its expression may also be observed in natural killer cells and certain populations of dendritic cells. Compared with CA, CD8+ cell density was 1.63-fold higher in BOT (*p* = 0.128), 2.68-fold higher in LGSC (*p* < 0.001), and 3.83-fold higher in HGSC (*p* < 0.001) (Figure 3 and Figure 4).

Comparative quantitative analysis of T-lymphocyte subpopulations in the peritumoral zone of serous ovarian neoplasms revealed marked differences in the density of the analyzed immune cell populations across histological groups, indicating substantial heterogeneity of the local immune microenvironment.

FOXP3 is an intranuclear transcription factor commonly associated with regulatory T lymphocytes (Tregs), although its expression may also be detected in rare fractions of transiently activated conventional T cells. Accordingly, quantitative assessment of FOXP3+ cells was used to characterize the relative abundance of a regulatory T-cell-associated population within the local immune microenvironment across the investigated histological groups.

Comparative quantitative analysis of FOXP3+ cells demonstrated a 2.75-fold higher density in BOT (*p* < 0.001), a 1.16-fold higher density in LGSC (*p* = 0.04), and a 9.07-fold higher density in HGSC compared with CA (*p* < 0.001) (Figure 3 and Figure 4). The highest FOXP3+ cell density was observed in HGSC, followed by BOT and LGSC, whereas the lowest values were recorded in CA.

Particular attention was paid to the distribution of T-lymphocytes in ovarian malignancies depending on pT1 substage. In a comparative analysis of the distribution of CD immunopositive cells in malignant neoplasms of various pT1 substages, we identified a numerical trend toward a gradual decrease in TIL density from pT1a to pT1c3; however, this pattern was not statistically significant. In HGSC, the densities of CD3+, CD4+, and CD8+ cells showed a numerical decrease from pT1a to pT1c3, whereas FOXP3+ cell density showed an opposite numerical trend, gradually increasing toward pT1c3. In LGSC, the densities of all analyzed immunopositive cell populations showed a numerical decrease across pT1 substages (Table 2).

To clarify the relationship between the immune microenvironment and the mutation profile in patients with ovarian serous neoplasms, a comprehensive analysis of the distribution density of immunopositive cells was performed in accordance with different types of mutations. The highest degree of lymphocytic infiltration was observed in tumors with DNA repair defects: BRCA1/2-mutated HGSC demonstrated the highest density of all analyzed immunopositive cell populations, while HRD-positive tumors were also characterized by relatively high lymphocyte densities in HGSC, LGSC, and BOT. At the same time, neoplasms associated with KRAS/BRAF mutations were characterized by comparatively lower densities of the analyzed immune markers. In LGSC and BOT, KRAS/BRAF-mutated tumors consistently demonstrated lower values of CD3+, CD4+, CD8+, and FOXP3+ cells. Interestingly, the only patient with KRAS/BRAF mutation from the CA group (*n* = 1) also demonstrated low density values for all analyzed immune cell populations; however, this observation was considered descriptive and was not used for subgroup-level conclusions. These patterns highlight differences in immune-cell infiltration across molecular subgroups and histological types of serous ovarian neoplasms (Table 3).

To further characterize the relative balance between CD8+ and FOXP3+ cell populations, we calculated the CD8+/FOXP3+ ratio. The CD8+/FOXP3+ ratio was used as a comparative index reflecting the relative abundance of CD8+ cells in relation to FOXP3+ cells. A comparative analysis of the index values revealed the highest CD8+/FOXP3+ ratio in the LGSC group. The minimum values were recorded in the HGSC cohort, indicating the lowest relative proportion of CD8+ cells to FOXP3+ cells among the analyzed groups. The BOT and CA groups occupied an intermediate position, while the index value in BOT was significantly lower than in the CA group (*p* = 0.014) (Table 4).

Thus, according to the quantitative analysis of the distribution of T-lymphocytes in serous epithelial neoplasia of various histological types, the highest densities of the analyzed immunopositive cells were observed in HGSC samples, with CD3+ cells representing the most abundant population. Along with this, the severity of lymphocytic infiltration varied according to the molecular genetic characteristics of the neoplasm: the highest densities of the analyzed immune cell populations were observed in BRCA1/2-mutated HGSC and HRD-positive subgroups. The analysis of cell distribution depending on the stage revealed a numerical trend toward a gradual decrease in the density of most analyzed TIL populations from pT1a to pT1c3; however, these differences did not reach statistical significance.

### 3.3. Age-Adjusted Multivariable Analysis of Immunopositive Cell Densities

**Figure 5:**
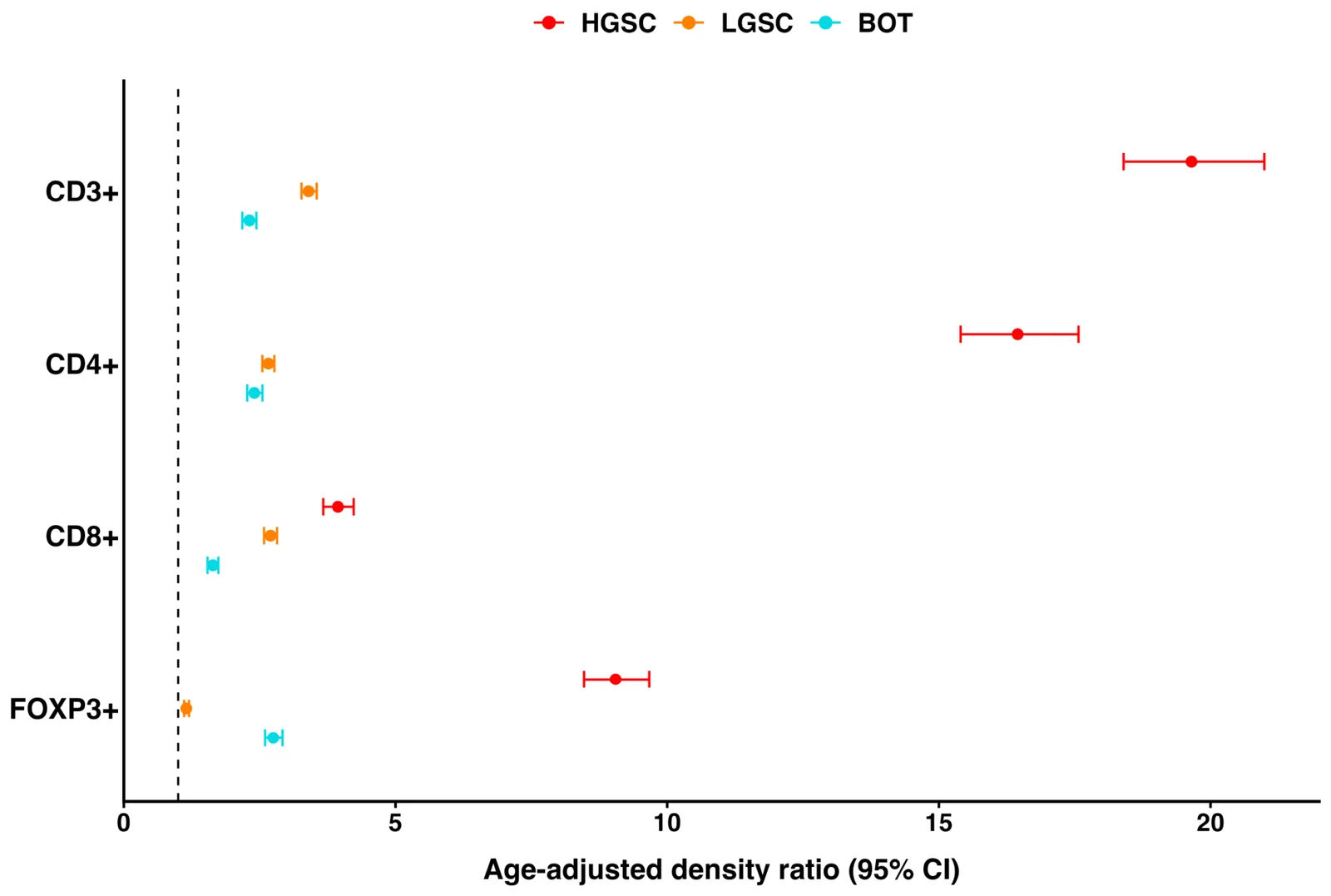
Age-adjusted density ratios of CD3+, CD4+, CD8+, and FOXP3+ immunopositive cells across histological groups, with CA as the reference category. Points and horizontal bars represent adjusted density ratios and 95% confidence intervals, respectively. The vertical dotted line at 1 represents the null effect for the density ratio.

After adjustment for patient age, the histological type of the neoplasm remained significantly associated with CD3+, CD4+, CD8+, and FOXP3+ cell densities (all *p* < 0.001). Compared with CA, the highest age-adjusted density ratios for all four markers were observed in HGSC, particularly for CD3+ and CD4+ cells. BOT and LGSC showed smaller but statistically significant age-adjusted differences relative to CA, while FOXP3+ cell density demonstrated a distinct pattern, with a higher adjusted ratio in BOT than in LGSC. All age-adjusted comparisons with CA were statistically significant (*p* < 0.001). Age itself was not independently associated with CD3+, CD4+, CD8+, or FOXP3+ cell density in the adjusted models (all *p* > 0.05) (Figure 5).

## 4. Discussion

In our study, a comprehensive immunohistochemical analysis of the characteristics of T-lymphocytic infiltrate of serous ovarian neoplasms of various histological subtypes was performed, as a result of which key immunophenotypic quantitative features were identified. The results obtained show that serous neoplasms of the ovary of women of both reproductive and menopausal age are characterized not only by differences in the degree of aggressiveness and mutation profile, but also by marked heterogeneity in the density and spatial distribution of T-lymphocytic populations within the local tumor microenvironment.

The observed shifts in the density of immune cells of the peritumoral zone and the tumor itself—CD3+ T-lymphocytes, CD4+ cells, CD8+ cells, and FOXP3+ cells—were associated with the histological type of the neoplasm and selected molecular characteristics, which is consistent with previously described heterogeneity of the immune microenvironment in ovarian serous neoplasms. [19,20,21,22].

Despite the established clinical relevance of TILs, previous studies indicate a limited number of detailed immunological and pathomorphological studies of this group of neoplasms in this context. To date, comparative data on immunohistochemical profiling of CD3+, CD4+, CD8+, and FOXP3+ cell subpopulations, strictly stratified by histological subtypes of ovarian serous neoplasms, remain very limited. Many available studies include mixed or generalized cohorts, which may obscure the biological and pathogenetic differences between the types of serous neoplasms developing through different pathways. A more detailed characterization of the immune profiles of individual histological subtypes may therefore contribute to a better understanding of their tumor microenvironment and provide a basis for further studies [19,21,22]. Quantitative and qualitative changes in the peritumoral immune landscape across serous ovarian neoplasms with different degrees of malignancy remain incompletely characterized. Similarly, the immunological patterns associated with indolent and more aggressive tumor phenotypes require further clarification. The lack of multicomponent profiling of these cellular subpopulations creates a significant gap in understanding differences in the tumor immune microenvironment, making pathomorphological stratification of the local immune response a useful framework for further investigation of immune remodeling during early tumor progression.

**Figure 6:**
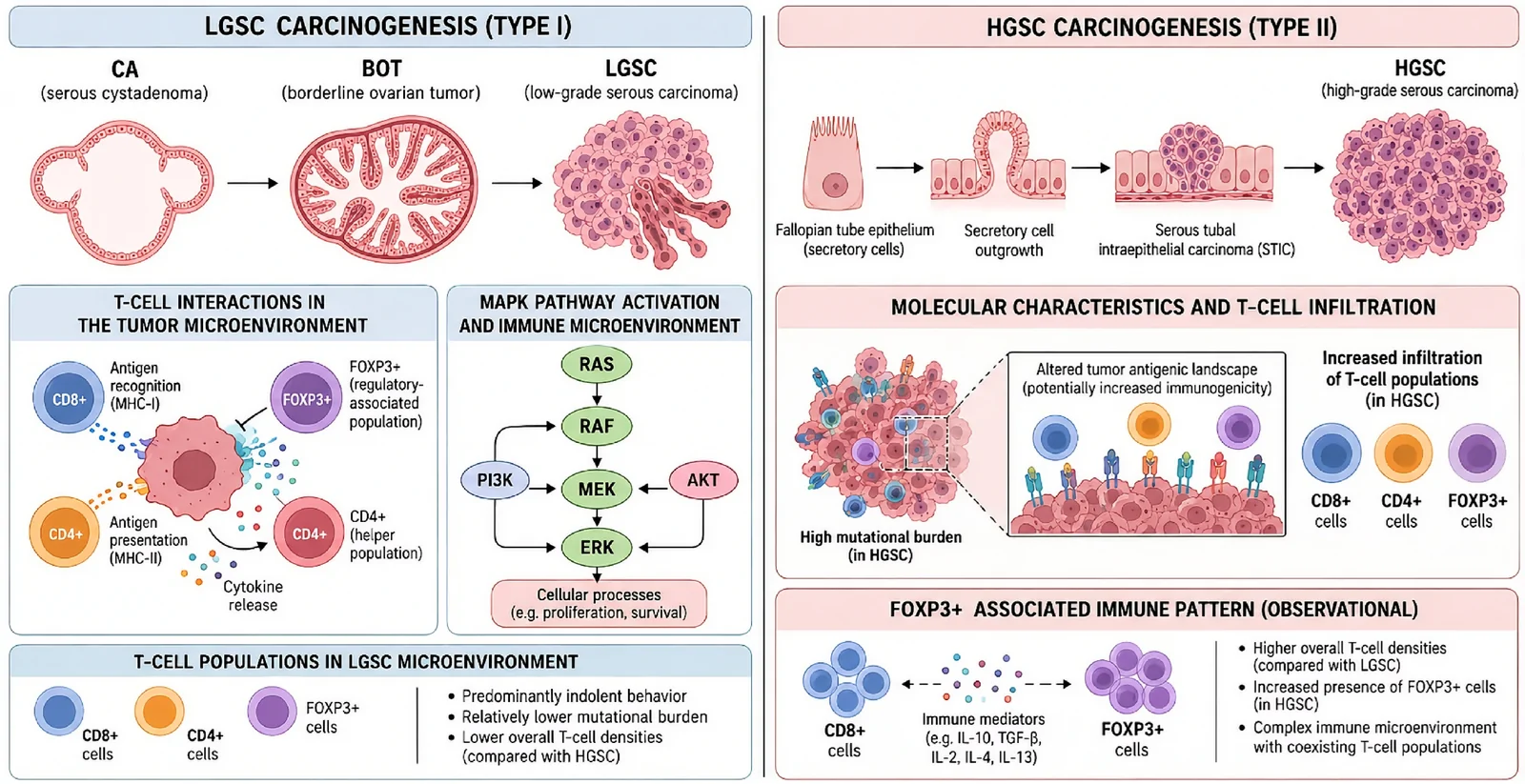
Conceptual representation of the observed immunophenotypic patterns and possible immune microenvironment remodeling in serous ovarian neoplasms.

The proposed mechanism of TILs involvement in the remodeling of the immune microenvironment in ovarian serous neoplasms is presented in Figure 6.

A useful framework for interpreting these findings is the dualistic model of ovarian carcinogenesis, which identifies two major pathways for the development of serous ovarian tumors. The evolution of type I neoplasms (low grade pathway) is commonly described as a morphologically continuous cascade. Initially, the single-layered cubic ciliated epithelium, devoid of cellular atypia, lining the benign CA, may undergo a gradual proliferative transformation. At the BOT stage, this leads to the formation of a complex hierarchical architecture of branching papillae, the loss of monolayer epithelial architectonics with the formation of cell clusters, and the rise of nuclear atypia in the strict absence of destructive invasion. Progression to LGSC is characterized histologically by true destructive stromal invasion, the formation of micropapillary growth patterns against the background of maintaining a relatively low mitotic index. At the molecular level, this process is associated with driver alterations in the MAPK signaling cascade, involving the RAS-RAF-MEK-ERK axis and its crosstalk with the PI3K and AKT pathways. Previous studies suggest that MAPK-related alterations may influence tumor-immune interactions; in this context, antigen presentation via MHC-I and recognition by TCR, together with cytokine-mediated recruitment of T-cell populations, represent possible components of the local immune response. These mechanisms may contribute to the distinct immune profiles observed across type I serous neoplasms; however, the present immunohistochemical data do not directly establish functional immunosuppression or blockade of T-cell effector activity at the BOT stage [21,22,23,24].

A completely different, most aggressive model of carcinogenesis is realized in the development of type Il neoplasms (high grade pathway). Most often, this pathway originates from the fallopian tube epithelium, rapidly progressing from normal secretory cells through precursor lesions, including serous tubal intraepithelial carcinoma (STIC), and ultimately to invasion and development of HGSC. The key molecular features associated with this pathway include genomic instability and alterations assessed in our study, including TP53, BRCA1/2, and HRD. These alterations may contribute to increased tumor immunogenicity and could partly explain the higher T-cell densities observed in HGSC; however, neoantigen burden was not directly assessed in the present study. Recent multimodal spatial profiling studies have also demonstrated immune microenvironment remodeling in fallopian tube precursors and HGSC. These findings support the possibility that regulatory and effector T-cell populations undergo substantial spatial and functional reorganization during tumor development, although the present immunohistochemical analysis does not directly establish cytokine-mediated suppression or functional exhaustion of CD8+ cells [21,22,24,25].

The presence of a high level of genomic instability is traditionally associated with the aggressive nature of the neoplasm and high proliferative activity, which is typical for HGSC [4]. However, the similar phenotype that we have recorded in the more indolent LGSC group requires a separation of the concepts of genetic instability and markers of tumor proliferative activity. This discrepancy suggests the need for a deeper study of the molecular changes underlying the formation of genomic alterations among tumors with a low degree of aggressiveness.

The absence of BRCA1/2 mutations in the cohorts of patients with LGSC and BOT does not exclude HRD. Previous studies indicate that impairment of the homologous recombination repair pathway may also be associated with epigenetic alterations, such as BRCA1 promoter hypermethylation, or with mutations in other homologous recombination genes (RAD51C, RAD51D, PALB2, BRIP1, BARD1) [26,27]. These defects may lead to genomic scar patterns, including LOH, TAI, and LST, forming an HRD phenotype in the absence of BRCA1/2 mutations.

Oncogene-induced replication stress may also contribute to genomic instability. Previous studies indicate that, unlike HGSC, in which TP53 alterations are highly characteristic, LGSC and BOT are more commonly associated with KRAS/BRAF pathway alterations [22,28].

The hyperactivation of proliferative signals induced by these mutations may lead to replication stress and subsequent double-strand DNA breaks in the early stages of tumor progression [29,30].

These data suggest that the genomic instability index should not be considered solely as a consequence of current impairment of DNA repair. Based on the fact that the vast majority of LGSCs and BOT studied by us exhibit a low Ki-67 index, we hypothesize that the HRD+ status in these tumors may, at least in part, reflect a persistent genomic scar rather than ongoing homologous recombination deficiency. One possible explanation is that such genomic alterations were accumulated during earlier periods of oncogene-induced replication stress associated with KRAS/BRAF pathway activation; however, this mechanism was not directly assessed in the present study [28,29,30].

According to the results of the analysis of immunohistochemical staining, an increase in the density of CD3−, CD4−, and CD8− immunopositive cells was observed across histological types with increasing malignant potential. On the one hand, this pattern may be associated with greater genomic instability and tumor immunogenicity; an increased neoantigen burden represents one possible mechanism described in the literature, although neoantigens were not directly assessed in the present study. On the other hand, an analysis of the distribution of CD3+ cells revealed their predominant location along the invasive edge of the tumor, which may reflect preferential T-cell accumulation at the tumor-stroma interface associated with invasive growth. This pattern of increasing T-cell infiltration with greater tumor immunogenicity and changes in the tumor-stromal interface is consistent with the findings of other authors [21,22,23,24,31,32,33].

Of particular interest is the sharp increase in the number of CD3+, CD4+, and CD8+ lymphocytes in the HGSC cohort recorded in our study, compared with a gradual, stepwise increase in the CA, BOT, and LGSC series. Such a pronounced difference is consistent with the classical dualistic model of ovarian carcinogenesis described above. Within the framework of this concept, type I tumors are characterized by a gradual transition and a relatively low mutational load, which may be associated with more moderate immune-cell infiltration. On the contrary, type II tumors, which develop through a distinct pathway and are not associated with type I, are characterized by marked genomic instability and a higher mutational burden. The resulting increase in tumor immunogenicity may contribute to more pronounced T-cell infiltration; however, neoantigen burden was not directly assessed in the present study [22,25]. Importantly, the association between histological type and CD3+, CD4+, and CD8+ cell densities remained significant after adjustment for patient age, whereas age itself was not independently associated with these parameters.

The data we have obtained may be of scientific and potential translational relevance with regard to the prognostic significance of the tumor microenvironment in malignant serous neoplasms, particularly in HGSC, although prognostic significance was not directly assessed in the present cohort. According to published data, increased intratumoral CD3+ and CD8+ lymphocytic infiltration has been associated with more favorable clinical outcomes, including longer survival [14,32,33,34,35].

However, the prognostic relevance of CD3+, CD4+, and CD8+ cell infiltration may differ between histological subtypes, and the available data for LGSC remain limited; therefore, the association between T-cell infiltration and overall survival in LGSC requires further validation [36].

Early-stage ovarian cancer is associated with substantially more favorable clinical outcomes, emphasizing the importance of understanding the biological changes occurring during early tumor progression. However, the evolution of the local immune microenvironment across early pT substages of serous ovarian carcinoma remains incompletely characterized. In our study, no statistically significant differences in TIL density were observed across pT1 substages, although numerical changes in the densities of individual T-cell populations were noted. For most T-cell populations, cell density tended to decrease with advancing pT1 substage from pT1a toward pT1c3, whereas FOXP3+ cells showed an opposite numerical tendency, with increasing density toward pT1c3. These observations highlight the need for further investigation of the relationships between T-cell distribution, local tumor progression, and molecular profiles during the early stages of serous ovarian carcinogenesis [3].

Eventually, the presented data extend the understanding of the pathomorphogenesis of serous ovarian neoplasia beyond classical descriptive morphology, indicating that differences in T-lymphocyte subpopulations represent an additional feature associated with tumor progression and histological subtype. The disclosure of the unique patterns of distribution of immunocompetent cells in relation to different carcinogenic pathways is consistent with the dualistic model of carcinogenesis at the immunological level, but does not by itself establish its validity or justify changes in diagnostic algorithms. Immunohistochemical profiling of CD3, CD4, CD8, and FOXP3 may provide a basis for further studies evaluating their potential prognostic and predictive relevance and their possible integration into individualized therapeutic approaches; however, prospective validation is required before these markers can be incorporated into routine practice or used to predict treatment response [21,25,31].

**Figure 7:**
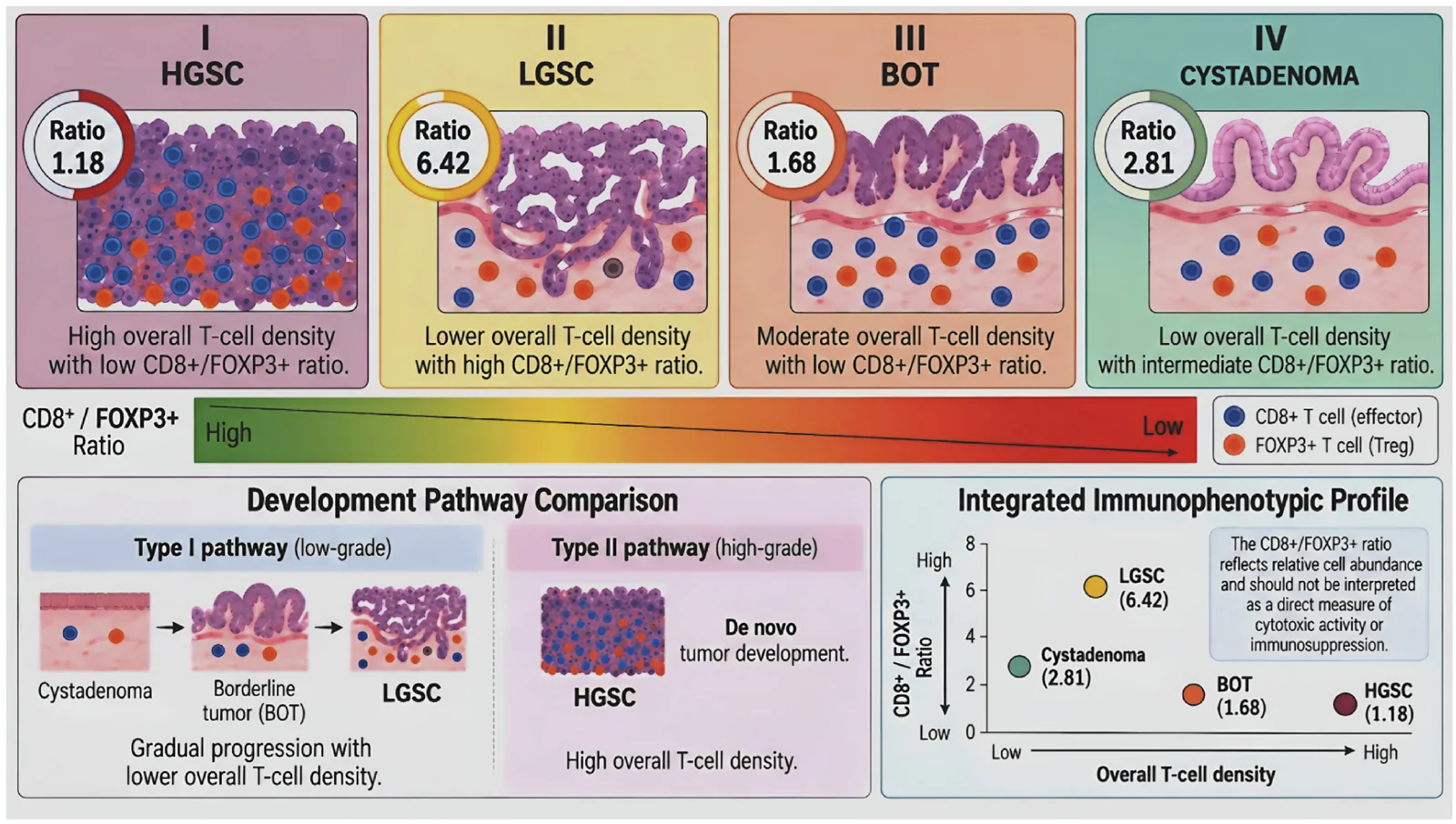
Schematic summary of immune remodeling in serous ovarian neoplasms.

One of the most intriguing results of our study is the revealed heterogeneity in the relative distribution of CD8+ and FOXP3+ cells across histological groups. The evaluation of the CD8+/FOXP3+ ratio demonstrated an unexpected pattern: the highest values of the ratio were recorded in LGSC and CA, whereas the lowest values were observed in the HGSC and BOT cohorts. Importantly, this ratio should be interpreted as a comparative index reflecting the relative abundance of CD8+ and FOXP3+ cells rather than as a direct measure of cytotoxic activity or local immunosuppression. The combination of high overall T-cell density with a low CD8+/FOXP3+ ratio in HGSC suggests a distinct immune-cell composition that may reflect complex immune remodeling, although the functional state of these populations was not directly assessed in the present study (Figure 7).

The most notable aspect of the immune profile observed in our study is the dramatic shift in ratio between BOT and LGSC. This phenomenon may reflect distinct patterns of immune remodeling within the type I pathway of serous ovarian carcinogenesis. In BOT, the lower CD8+/FOXP3+ ratio indicates a relatively greater abundance of FOXP3+ cells in relation to CD8+ cells, whereas LGSC demonstrates a substantially higher ratio. Given the well-established association of LGSC with KRAS/BRAF-driven MAPK pathway alterations, these differences may be related to changes in tumor-immune interactions accompanying type I carcinogenesis. Taken together, these findings support the hypothesis that the BOT–LGSC spectrum is accompanied by substantial remodeling of the local immune microenvironment, although the functional mechanisms underlying this shift require further investigation. [22,23,35,37,38].

The potential clinical relevance of the results obtained lies in the fact that the identified patterns of TIL distribution may help define histology-specific immune profiles of serous ovarian neoplasms and provide a rationale for further therapeutic investigation. In HGSC, the combination of high overall T-cell density with a low CD8+/FOXP3+ ratio indicates a distinct immune-cell composition; however, these findings do not directly demonstrate functional immunosuppression or predict response to immunotherapy. In light of previous studies investigating combined PD-1/PD-L1 and CTLA-4 blockade in ovarian cancer, the immune profile observed in HGSC in our cohort may identify this histological subtype as a biologically plausible candidate for further evaluation of combined immune-checkpoint strategies. Nevertheless, this hypothesis requires prospective and functional validation before it can be translated into treatment selection [39].

In contrast, the indolent course of LGSC was characterized by lower T-cell densities, suggesting a distinct pattern of immune-cell distribution within the tumor microenvironment. The biological significance of this pattern remains to be clarified and may be related to differences in tumor-immune interactions associated with the type I pathway of serous ovarian carcinogenesis. In the broader context of immune checkpoint-based approaches, including the PD-1/PD-L1 blockade investigated in ovarian cancer, such differences in immune-cell composition may warrant further study; however, the present data do not allow conclusions regarding treatment sensitivity or selection of patients for checkpoint inhibitor therapy [39,40]. Further integration of immunohistochemical profiles with patient survival and treatment-response data may help determine their potential prognostic and predictive relevance. However, such clinical applications require validation in studies incorporating longitudinal clinical outcomes before these markers can be considered for routine use.

Besides the immunological differences described above, the histological groups also differed in age distribution. Our analysis of the demographic characteristics of the studied groups revealed a clear age gradient across histological types, with HGSC represented by an older patient group than CA and BOT. These differences are consistent with the distinct clinical and pathogenetic characteristics of the studied neoplasms. The indolent type of tumor progression, encompassing CA, BOT, and LGSC, was observed predominantly in younger patients than HGSC in our cohort. Such age-related differences may be relevant to clinical management, particularly when reproductive considerations are taken into account [2]. However, the present study was not designed to evaluate fertility-preserving strategies or treatment selection. On the contrary, HGSC was characterized by a marked shift in the age of detection towards an older age group. Although this age distribution is clinically relevant, it should not be interpreted as a direct determinant of treatment strategy. Thus, the revealed age-associated heterogeneity highlights the importance of considering patient age and clinical context when interpreting differences between histological types, whereas the potential clinical value of immune profiling requires further validation in studies incorporating treatment and reproductive outcomes.

Thus, based on this study, it can be concluded that the results obtained demonstrate the immune heterogeneity of ovarian serous neoplasms, with differences in the densities of CD3+, CD4+, CD8+, and FOXP3+ cells reflecting distinct patterns of the immune component of the tumor microenvironment. These findings may provide a basis for further refinement of pathomorphological assessment and investigation of the potential clinical relevance of immune profiling.

Finally, the results of the study support further development of comprehensive approaches to the diagnosis and assessment of serous ovarian neoplasms. An in-depth characterization of the immune landscape of these tumors, from the lower-density immune profiles observed in indolent neoplasms to the pronounced T-cell infiltration and distinct CD8+/FOXP3+ ratios observed in more aggressive histological types, provides a framework for more precise characterization of their immune phenotypes. Quantitative assessment of the local immune response may therefore contribute to the further refinement of pathomorphological stratification and, when integrated with clinical outcome data, may help clarify the potential relevance of these immune patterns to prognosis and treatment response. Future studies incorporating survival, treatment-response, and reproductive outcomes will be important to determine whether TIL profiles may ultimately contribute to individualized clinical management.

## 5. Limitations

This study has several limitations. First, our cohort was restricted to early-stage disease. Although this provided an opportunity to characterize the local immune microenvironment in early-stage serous ovarian neoplasms, it limits the extrapolation of the observed immunological patterns to advanced and metastatic stages.

Secondly, we relied on standard immunohistochemistry, with the immune profiling focused primarily on the T-cell compartment. This approach limits a more comprehensive assessment of cellular interactions within the tumor microenvironment and leaves other potentially important immune populations, such as tumor-associated macrophages and B-lymphocytes, insufficiently characterized. These components could be further investigated in future studies using multiplex immunofluorescence approaches.

Thirdly, a reliable assessment of progression-free survival was not possible within the available follow-up period, since patient recruitment into the cohort was completed in mid-2025, resulting in an insufficient follow-up period for reliable assessment of relapse and long-term clinical outcomes. Therefore, the prognostic and treatment-response significance of the observed immune profiles could not be directly assessed in the present study and requires further evaluation in studies with longer follow-up.

Fourth, our targeted molecular profiling of selected recurrent mutational hotspots limits the ability to capture the full landscape of genomic alterations. In the future, comprehensive genomic profiling may provide a more complete characterization of genomic instability and help clarify the relationships between molecular alterations, potential neoantigen burden, and the immune microenvironment.

Fifth, the retrospective single-center design and unequal sizes of the histological groups, together with small numbers within some molecular subgroups, may introduce selection bias, limit the statistical power of subgroup comparisons, and reduce the generalizability of the findings to other patient populations and clinical settings.

It should be emphasized separately that this article is primarily devoted to the comparative characterization of the immune microenvironment of serous ovarian neoplasms, both malignant and benign, based on the distribution of selected immune-cell populations. However, the study does not directly assess the functional mechanisms underlying these immune patterns or the interactions between individual cellular and molecular components of the tumor microenvironment. Understanding these mechanisms will require further studies incorporating functional, spatial, and multiplex approaches to better characterize the biological significance of the observed immune profiles.

## 6. Conclusions

The results of this study demonstrate that serous ovarian neoplasms exhibit distinct histology-associated patterns of T-cell infiltration, with differences in CD3+, CD4+, CD8+, and FOXP3+ cell densities and in the CD8+/FOXP3+ ratio. HGSC showed the highest T-cell densities and the lowest CD8+/FOXP3+ ratio, while BRCA1/2-mutated and HRD-positive tumors showed comparatively high T-cell densities. These histology-associated differences remained significant after adjustment for patient age, whereas no statistically significant differences in TIL density were observed across pT1 substages.

Further studies integrating multiplex and functional approaches with longitudinal clinical outcomes are needed to determine the biological, prognostic, and predictive significance of these immune profiles.

## Figures and Tables

**Table 1:** Demographic and molecular characteristics of patients with serous ovarian neoplasms.

Characteristics	HGSC (*n* = 108)	LGSC (*n* = 49)	BOT (*n* = 16)	CA (*n* = 54)
Age, years (mean ± SD)	59.7 ± 3.7	46.9 ± 4.3	45.3 ± 2.6	43.6 ± 3.9
Mutations & Markers, *n* (%)				
*TP53*	90 (83.3%)	—	—	—
*BRCA1/2*	24 (22.2%)	—	—	—
*KRAS/BRAF*	—	30 (61.2%)	9 (56.3%)	1 (1.9%)
HRD+	45 (41.7%)	22 (44.9%)	7 (43.8%)	—
High Ki-67	104 (96.3%)	—	—	—
Low Ki-67	—	46 (93.9%)	14 (87.5%)	—

**Table 2:** Density of distinct subpopulations of TILs across early pathological substages of malignant serous ovarian neoplasms.

Stage	CD3+ (Me [Q1–Q3])	CD4+ (Me [Q1–Q3])	CD8+ (Me [Q1–Q3])	FOXP3+ (Me [Q1–Q3])
**HGSC, *n* = 108**				
pT1a (IA), *n* = 11	1819.24 [1777.53–1859.61]	884.82 [868.32–893.42]	147.30 [143.96–152.56]	101.42 [97.59–105.74]
pT1b (IB), *n* = 8	1744.31 [1731.27–1763.53]	869.32 [855.82–878.22]	142.32 [140.34–145.73]	105.80 [104.29–108.07]
pT1c1 (IC1), *n* = 19	1704.42 [1673.91–1728.18]	844.62 [836.62–852.52]	138.15 [134.36–141.56]	109.01 [107.02–111.42]
pT1c2 (IC2), *n* = 44	1637.88 [1593.26–1669.44]	808.94 [789.12–824.82]	131.40 [127.04–135.38]	113.69 [110.97–116.65]
pT1c3 (IC3), *n* = 26	1525.29 [1478.12–1569.78]	748.92 [735.02–764.02]	123.13 [117.96–125.78]	120.52 [115.86–125.24]
**LGSC, *n* = 49**				
pT1a (IA), *n* = 7	353.49 [340.63–367.18]	158.99 [150.50–166.16]	110.65 [105.56–114.93]	16.82 [16.00–17.70]
pT1b (IB), *n* = 6	329.96 [323.32–337.23]	147.60 [144.05–152.25]	103.69 [99.73–106.62]	16.08 [15.60–16.45]
pT1c1 (IC1), *n* = 9	312.04 [302.84–318.64]	139.39 [133.62–142.12]	97.67 [88.99–100.83]	15.07 [14.40–15.64]
pT1c2 (IC2), *n* = 16	286.78 [277.63–295.35]	129.17 [124.64–134.39]	91.04 [83.63–94.16]	13.81 [13.40–14.40]
pT1c3 (IC3), *n* = 11	251.30 [236.26–261.58]	113.10 [105.10–119.71]	78.66 [73.95–87.43]	12.05 [11.41–12.72]

**Table 3:** Density of distinct TILs subpopulation stratified by selected molecular characteristics across the histopathological spectrum of serous ovarian tumors.

Characteristics	CD3+ (Me [Q1–Q3])	CD4+ (Me [Q1–Q3])	CD8+ (Me [Q1–Q3])	FOXP3+ (Me [Q1–Q3])
**HGSC, *n* = 108**				
*TP53* mutation, *n* = 90	1642.52 [1581.32–1704.87]	814.22 [773.32–849.82]	132.72 [124.27–140.34]	112.47 [107.47–116.65]
*BRCA 1/2* mutation, *n* = 24	1749.11 [1716.27–1792.56]	866.32 [848.52–882.22]	143.49 [138.78–151.01]	120.52 [117.47–125.24]
HRD+, *n* = 45	1695.33 [1648.43–1737.62]	841.92 [818.82–864.42]	138.81 [133.11–146.25]	116.61 [113.31–121.21]
High Ki-67, *n* = 104	1646.68 [1584.61–1707.12]	815.52 [775.92–851.22]	133.47 [125.78–141.56]	112.51 [107.84–117.48]
**LGSC, *n* = 49**				
*KRAS/BRAF* mutation, *n* = 30	278.19 [258.45–293.86]	124.13 [114.98–130.44]	86.73 [78.66–93.85]	13.40 [12.41–14.07]
HRD+, *n* = 22	327.11 [314.18–344.38]	145.11 [136.78–154.13]	103.66 [97.67–109.12]	16.05 [15.13–16.82]
Low Ki-67, *n* = 46	294.85 [269.87–318.61]	132.07 [121.50–143.10]	92.61 [84.47–101.98]	14.28 [13.04–15.44]
**BOT, *n* = 16**				
*KRAS/BRAF* mutation, *n* = 9	183.81 [164.80–197.73]	110.15 [98.61–117.15]	51.54 [44.70–56.36]	31.65 [27.62–33.91]
HRD+, *n* = 7	228.56 [216.22–246.01]	136.31 [125.39–147.02]	63.77 [58.67–68.78]	38.25 [35.67–41.70]
Low Ki-67, *n* = 14	197.73 [177.32–216.22]	121.27 [106.23–134.35]	56.36 [50.63–61.94]	34.23 [30.93–37.54]
**CA, *n* = 54**				
*KRAS/BRAF* mutation, *n* = 1	65.65	35.09	24.57	9.03

**Table 4:** CD8+/FOXP3+ ratio in serous ovarian tumors.

Cohort	CD8+/FOXP3+ Ratio (Me [Q1–Q3])
**HGSC**	1.18 [1.06–1.34]
**LGSC**	6.42 [5.85–7.15]
**BOT**	1.68 [1.38–2.01]
**CA**	2.81 [2.44–3.19]

## Data Availability

No new data were created or analyzed in this study. Data sharing is not applicable to this article.

## Abbreviations

The following abbreviations are used in this manuscript:

AJCC	American Joint Committee on Cancer
BOT	Borderline ovarian tumor
CA	Cystadenoma
DAB	3,3′-diaminobenzidine
ECOG	Eastern Cooperative Oncology Group
FFPE	Formalin-fixed paraffin-embedded
FIGO	International Federation of Gynecology and Obstetrics
H&E	Hematoxylin and eosin
HGSC	High-grade serous ovarian carcinoma
HRD	Homologous recombination deficiency
ICD-10	International Classification of Diseases, 10th Revision
IHC	Immunohistochemistry
LGSC	Low-grade serous ovarian carcinoma
LOH	Loss of heterozygosity
LST	Large-scale state transitions
MHC	Major histocompatibility complex
NGS	Next-generation sequencing
OC	Ovarian cancer
ROI	Region of interest
PCR	Polymerase chain reaction
TAI	Telomeric allelic imbalance
TCR	T-cell receptor
TILs	Tumor-infiltrating lymphocytes
TIME	Tumor immune microenvironment
TLA	Three-letter acronym
TMB	Tumor mutational burden
TME	Tumor microenvironment
Tregs	Regulatory T-cells
WHO	World Health Organization
